# Characterization of putative pathogenic *Shewanella algae* isolated from ballast water

**DOI:** 10.14202/vetworld.2021.678-688

**Published:** 2021-03-19

**Authors:** Nik Nuraznida Nik Ibrahim, Nurathirah Mat Nasir, Fathul Karim Sahrani, Asmat Ahmad, Fareed Sairi

**Affiliations:** 1 Department of Biological Sciences and Biotechnology, Faculty of Science and Technology, Universiti Kebangsaan Malaysia, 43600 Bangi, Selangor, Malaysia; 2 Department of Earth Sciences and Environment, Faculty of Science and Technology, Universiti Kebangsaan Malaysia, 43600 Bangi, Selangor, Malaysia

**Keywords:** ballast water, extracellular enzymes, putative pathogen, *Shewanella algae*

## Abstract

**Background and Aim:**

*Shewanella algae* is ubiquitous in marine-associated environments and has been increasingly recognized as a significant human pathogen that can cause serious infections mainly associated with exposure to seawater and ingestion of raw seafood. This study aimed to isolate and characterize *S. algae* from ballast water of ships berthed at Port Klang, Malaysia.

**Materials and Methods:**

Ballast water was sampled from nine ships docked at Port Klang, Malaysia. The isolates were identified and characterized based on biochemical and enzymatic properties, *16S rRNA* and *gyrB* sequencing, biofilm formation capability, and antibiotic susceptibility.

**Results:**

A total of four *S. algae* isolates were isolated from four ballast water samples tentatively name Sa-BW1, Sa-BW2, Sa-BW7, and Sa-BW8. All isolates showed positive reaction for cytochrome oxidase, catalase, high tolerance to NaCl (6% and 8%), ability to grow at 42°C, and on *Salmonella-Shigella* agar. The strains also exhibited b-hemolytic activity on sheep blood and human blood agar, positive reaction for lipase, protease, DNase and gelatinase, strong biofilm adherence capabilities and multiple antibiotic resistances against ampicillin, carbenicillin, cephalothin, colistin, novobiocin, oxacillin, penicillin, rifampicin, and tobramycin which suggested their potential pathogenicity.

**Conclusion:**

This study demonstrated the occurrence of putative pathogen *S. algae* in ballast water of ships docked at Malaysian port.

## Introduction

Ships carry ballast water to control their stability and trim at the start of the voyage. However, the discharge of ballast water near ports risks the transport of invasive species, including pathogenic species from one continent to another. Should a novel genotype of pathogenic species arrived in ballast water, it may establish and persist in the local waters [1] which may lead to the emergence of new virulence strains in the existing population. The global distribution of pathogenic bacteria implicates a negative effect on the existing ecosystem as well as marine animal and human health. The concern about the transmission of potentially pathogenic bacteria through ballast water began in 1992, when the Centers for Disease Control and Prevention of the USA detected *Vibrio cholerae* in shellfish collected from ballast tanks of cargo ships that had come from South America [2]. Since then, the list of pathogenic species detected in ballast water has increased, including *Listeria monocytogenes*, *Mycobacterium* spp., several species of *Aeromonas*, *Pseudomonas*, *Vibrio*, *Staphylococcus*, the coral pathogens *Serratia marcescens*, and *Sphingomonas* spp. and some emerging opportunistic pathogen such as *Stenotrophomonas maltophilia* and *Shewanella algae* [3-5]. However, no discharge limits have been set for these pathogens.

*Shewanella* spp. is ubiquitous in marine-associated environments and widely spread in nature throughout the world. It is a facultative anaerobe, motile, and Gram-negative bacillus belonging to the family Shewanellaceae, order *Alteromonadales*. The physiological diversity and broad respiratory versatility of *Shewanella* spp. make it a highly adaptable organism that can survive in different environmental niches. In the previous studies, *Shewanella* spp. has been isolated from a wide range of environment including freshwater [6], estuary [7], deep sea [8], oil field [9], muddy sediment [10], fish [11], and marine sponge [12]. While members of this genus have been intensively studied for their role in bioremediation [13] and application in microbial fuel cells [14], several species have been reported as emerging pathogens in human and aquatic animals. Out of more than 60 known *Shewanella* species, *Shewanella ­putrefaciens*, *Shewanella haliotis*, *Shewanella xiamenensis*, and *S. algae* have been documented with pathogenicity in human beings [15-17]. Although human infection by these species is rare, increasing number of cases has been reported worldwide with >80% of clinical *Shewanella* isolates being attributed to the *S. algae* species. [18].

*S. algae* has been recognized as a conditionally pathogenic bacteria to human and aquatic animals [19,20]. The most common clinical symptoms described in human infections by *S. algae* are bacteremia, cellulitis, and chronic otitis media [18]. Rare cases of necrotizing soft-tissue infection [21], rupture of aortic aneurysm [22], peritonitis [23], and endocarditis was reported by Davidson *et al*. [24]. As for aquatic species, *S. algae* has been identified as a causative agent of ulcer disease for marine fish species, *Scinenops ocellata* and abalone, *Haliotis diversicolor* [11,25]. Other studies showed that *S. algae* can cause black spot disease to farmed freshwater shrimp, *Penaeus vannamei* and lesion in reared tonguefish, and *Cynoglossus semilaevis* [19,26]. The pathogenic potential of *Shewanella* has been controversial as most cases of human infection develop in people with underlying comorbidities and occurred as polymicrobial infections [18,27]. However, prior reports of monomicrobial infection with *S. algae* have suggested the organism as causal in many cases, confirming its ability to cause disease [28-30]. Although infection in healthy hosts is uncommon, rare cases have been reported in individuals with no underlying diseases due to massive exposure to marine environment and seafood consumption [31-33]. The increased in *S. algae* virulence was attributed to its hemolytic activity, enzymatic activity, and biofilm formation [18,34,35].

This study aimed to characterize *S. algae* strains isolated from ships’ ballast water incoming to Port Klang, Malaysia. The characterizations were carried out based on the biochemical and enzymatic properties, *16S rRNA* and *gyrB* gene sequence analysis, biofilm formation ability, and susceptibility to antibiotics.

## Materials and Methods

### Ethical approval and Informed consent

No ethical approval was required in this study. The sheep blood for preparation of sheep blood agar was kindly provided by UKM Animal House. The handling and collection of sheep blood were conducted by an assistant veterinary officer. While for human blood agar preparation, human blood samples were collected from healthy volunteers by a medical laboratory technician of the UKM Health Center. Informed consent was obtained from the volunteers included in the study. There was no direct involvement during the blood withdrawal procedures and no further experiments were performed on the subjected animal and human.

### Study period and location

This study was conducted from August 2016 to March 2017 in Port Klang, Malaysia, where ballast water samples were taken from several ships calling at Port Klang.

### Sample collection

Ballast water samples were taken from nine ballast tanks of nine-unit ships docked at Port Klang, Malaysia. The samples were collected using 20 L bucket through the manhole or overflow pipe when manhole was inaccessible which later filled into 1 L sterilized Schott bottle. Seawater samples were also taken from four sampling points of surrounding port water using Niskin water sampler. The samples were then transferred back to the laboratory in an icebox for further microbiological analysis. Details of each ballast water samples are shown in Table-1.

**Table-1:** Details of ballast water sampling.

Sample No.	Type of vessel	BW source/Port of origin	Location of sampling
BW1	Container ship	Singapore	Manhole
BW2	Container ship	Yokkaichi	Manhole
BW3	Container ship	Mundra	Sounding pipe
BW4	Container ship	Hong Kong	Manhole
BW5	Container ship	Ning Bo	Manhole
BW6	Container ship	Mormugao	Manhole
BW7	Container ship	Nhava Sheva	Manhole
BW8	Bulk carrier	Malacca strait	Manhole
BW9	Bulk carrier	Zhang Jia Gang	Manhole

### Isolation and selection of *Shewanella* spp.

The water samples were serially diluted up to 10^−5^ using 0.8% saline water and 0.1 mL of the diluted samples were inoculated on marine agar by spread plate technique. The plates were incubated at 30°C for 3 days. Different colonies were picked and purified by subculturing on marine agar for several times. Pure colonies with distinct morphologies were tested for the following key characteristics of *Shewanella*, according to the description by Holt and Bruun [18]: Gram stain, motility, cytochrome oxidase, catalase reaction (3% H_2_O_2_), and H_2_S production using sulfide indole motility medium. Colonies that fit the description of being Gram-negative bacillus, oxidase-catalase positive, and H_2_S producing were subjected to further tests.

### Biochemical and enzymatic activities

All presumptive *Shewanella* isolates were tested for: Utilization of glucose, sucrose, lactose, maltose and citrate, IMViC test (indole, Methyl red, Voges–Proskauer and citrate) nitrate reduction, growth at 4°C and 42°C, growth in 6% and 8% NaCl, and growth on *Salmonella-Shigella* (SS) agar and MacConkey agar. The following enzymatic activities were also determined: Lipase, protease, DNase, and gelatinase which were performed on spirit blue agar, skim milk agar, DNase agar, and gelatine medium, respectively. Hemolytic activity was determined by streaking the strains on heart infusion agar supplemented with 5% washed erythrocytes of human and sheep blood. The incubation was performed at 30°C for up to 72 h and the results were recorded.

### Confirmation of *S. algae* by *16S rRNA* and *gyrB* gene sequencing

Total genomic DNA of the presumptive *S. algae* isolates was extracted using CTAB/NaCl method [36]. The *16S rRNA* gene was amplified using universal primer, 27F (5’-AGAGTTTGATCCTGGCTCAG-3’) and 1492R (5′-TACGGCTACCTTGTTACGACTT-3′) [37]. A 25 mL polymerase chain reaction (PCR) mixture was prepared which consist of 1 mL genomic DNA, 12.5 mL 2 × PCR Master Dye Mix, 1 mL (0.1 mM) primer and 9.5 mL sterile distilled water. The PCR conditions were as follows: Initial denaturation at 94°C for 5 min, followed by 30 cycles of 94°C for 1 min, 55°C for 1 min, and 72°C for 2 min, with a final extension at 72°C for 10 min.

Amplification of *gyrB* gene was carried out using two degenerated primers, UP1 (5’-GAA GTCATCATGACCGTTCTGCAYGC NGGNGGNAARTTY GA-3’) and UP2R (5’-AGC AGGGTACGATGTGCGAGCCRTCNACRTCN GCRTCNGTCAT-3’) with the following conditions: Initial denaturation at 94°C for 1 min, followed by 30 cycles of denaturation at 94°C for 1 min, primer annealing at 60°C for 1 min, primer extension at 72°C for 2 min, and final extension at 72°C for 7 min [38].

Sequencing of the *16S rRNA* and *gyrB* amplicons was completed by a sequencing company (Apical Scientific Sdn Bhd, Selangor, Malaysia). The obtained sequence data were aligned and analyzed using BioEdit Version 7.0. Species identification was performed by Basic Local Alignment Search Tool (BLAST) (http://blast.ncbi.nlm.nih.gov/Blast.cgi).

### Phylogenetic analysis

The phylogenetic relationship of *S. algae* was determined by comparing the obtained *16S rRNA* and *gyrB* gene sequences with highly identical existing sequences in GenBank database using the BLAST algorithm. Sequences of other *Shewanella* species were also collected as comparison to the *S. algae*. The multiple alignment and construction of neighbour-joining (NJ) and maximum likelihood (ML) phylogenetic trees were performed using MEGA Version 6.0 [39]. The topology of the phylogenetic tree was evaluated by a bootstrap analysis through 1000 replications. *Escherichia coli* was used as an outgroup.

### Biofilm formation potential test

The ability of *S. algae* isolates to form biofilm was tested based on microtiter dish biofilm formation assay adapted from O’Toole [40]. The biofilm formation by the isolates was quantified based on the absorbance (OD) reading at 595 nm. Negative control wells contained only marine broth were added in the assay, while suspension of *Staphylococcus aureus* from UKM Biotechnology and Marine Microbiology Laboratory culture collection was included as positive control. The adherence capabilities of the isolates were classified according to Stepanović *et al*. [41]. The cutoff OD (OD_C_) was defined as three standard deviations above the mean OD of the negative control. In this study, the OD mean of the negative control was 0.083±0.02. Hence, the cutoff OD (OD_C_) applied in this study was set as 0.143. Comparing the OD means of isolates well to OD_c_ = 0.143, 2 × OD_c_ = 0.286 and 4 × OD_C_ = 0.332, the strains were then categorized as follows:

1. OD ≤ OD_C_ = Non adherent
2. OD_C_ < OD ≤ 2 × OD_C_ = Weakly adherent
3. 2 × OD_C_ < OD ≤ 4 × OD_C_ = Moderately adherent
4. 4 × OD_C_ < OD = Strongly adherent

### Antibiotic susceptibility test

Susceptibility of *S. algae* isolates to antibiotics was determined by Kirby-Bauer disk-diffusion technique on Mueller-Hinton agar following protocol by Hudzicki [42] using broad spectrum antibiotics used to treat variety of bacterial infections. The disks used were ampicillin (20 μg), carbenicillin (100 μg), cephalothin (30 μg), chloramphenicol (30 μg), ciprofloxacin (10 μg), colistin (10 μg), gentamicin (10 μg), kanamycin (30 μg), novobiocin (30 μg), oxacillin (5 μg), oxytetracycline (30 μg), penicillin (10 μg), rifampicin (5 μg), streptomycin (25 μg), and tobramycin (10 μg). The diameter of inhibition zone around each antibiotic disk was measured to the nearest millimeter. The zone diameters of each drug were interpreted using the criteria published by the Clinical and Laboratory Standards Institute. The strains were classified as susceptible (S), resistant (R), and intermediate (I) depending on the size of zone inhibition. Multiple antibiotic resistance (MAR) index was calculated as ratio of the number of antibiotics to which the test isolate depicted resistance to the total number of antibiotics to which the test isolate had been evaluated for susceptibility [43]. The MAR index was used as a tool to assess the risk of the isolates coming from a region of high or low antibiotic use. A MAR index >0.2 indicates a “high-risk” source of contamination.

## Results

### Characterization and identification of *S. algae*

A total of 102 isolates were successfully isolated from ballast water. Four of the isolates were presumed as *Shewanella* species, namely, Sa-BW1, Sa-BW2, Sa-BW7, and Sa-BW8. As for seawater samples, out of 212 isolates that were isolated two of them were presumed as *Shewanella*, namely, Sa-SW2 and Sa-SW3. The presumptive *Shewanella* isolates presented as Gram-negative rods and displayed positive reaction for oxidase-catalase test and production of H_2_S. All isolates appeared as circular orange colonies on marine agar. Further biochemical tests revealed that all the presumptive *Shewanella* spp. isolates were tolerant to high NaCl concentration (6%, and 8%) and able to grow at 42°C but not at 4°C. Negative reactions were observed for indole, methyl-red, Voges-Proskauer, and citrate tests. All strains reduced nitrate to nitrite and produced acid from glucose but not from sucrose, maltose, and lactose. The isolates also grew well on MacConkey and SS agar. Positive enzymatic activities for lipase, protease, DNase, and gelatinase were detected from all strains. They also exhibited a clear zone of beta hemolysis on sheep blood and human blood agar after 48 h incubation.

Comparison of the biochemical and enzymatic profiles displayed by the presumptive *Shewanella* isolates with other *Shewanella* type strains (*S. algae* IAM 14159, *S. putrefaciens* ATCC 8071, and *S. haliotis* JCM 14758), as shown in Table-2, suggested all strains as *S. algae*. Further identification by *16S rRNA* and *gyrB* gene sequencing confirmed the identity of the ballast water and seawater isolates. Nucleotide BLAST search of each strain showed >99% identity score to existing *S. algae* sequences in database. Sequence data of *S. algae* isolates of ballast water samples from this study were registered in GenBank under accession numbers: *S. algae* strain BW1 (16S; MN548355, *gyrB*; MN555561), *S. algae* strain BW2 (16S; MN548356, *gyrB*; MN555562), *S. algae* strain BW7 (16S; MN548357, *gyrB*; MN555563), and *S. algae* BW1 (16S; MN548358, *gyrB*; MN555564).

**Table-2:** Biochemical and enzymatic profiles of *S. algae* strains from ballast water.

Strain/Characteristics	*S. algae* (Sa-BW)	*S. algae* IAM 14159^T^	*S. putrefaciens* ATCC 8071^T^	*S. haliotis* JCM 14758^T^
Morphological characteristics				
Gram stain	−	−	−	−
Cell shape	rod	rod	rod	rod
Motility	+	+	+	+
Spores	−	−	−	−
Biochemical characteristics				
Oxidase	+	+	+	+
Catalase	+	NA	+	+
Voges–Proskauer	−	NA	NA	NA
Methyl red	−	NA	NA	NA
Indole production	−	NA	−	−
H2S production	+	+	+	+
Nitrate reduction	+	+	+	+
Utilization of glucose	+	+	+	−
Utilization of sucrose	−	−	−	−
Utilization of lactose	−	−	−	−
Utilization of maltose	−	−	−	−
Utilization of citrate	−	−	−	+
Growth on SS agar	+	+	−	NA
Growth on MacConkey	+	NA	NA	NA
Growth at 42°C	+	+	−	+
Growth at 4°C	−	−	+	−
Growth in 6% NaCl	+	+	−	+
Growth in 8% NaCl	+	NA	NA	+
Enzymatic characteristics:				
Lipase	+	+	+	−
Protease	+	NA	+	+
DNase	+	+	+	+
Gelatinase	+	+	NA	+
Hemolysis (sheep blood)	+	+	−	NA
Hemolysis (human blood)	+	NA	NA	NA

T =Type strain, NA=Not available, +=positive reaction, –=negative reaction. *S. algae*: *Shewanella algae*

### Phylogenetic analysis

**Figure-1:**
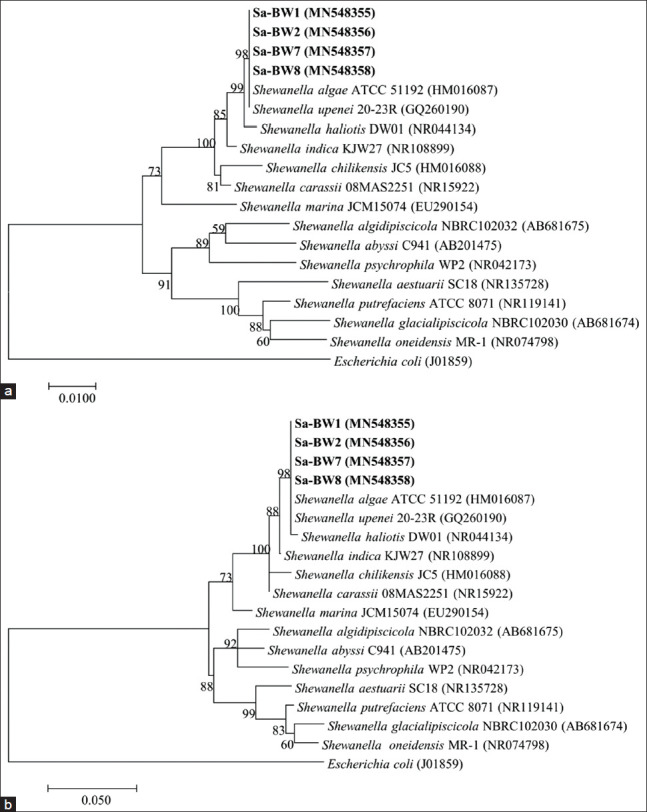
Phylogenetic tree based on *16S rRNA* gene sequences of *S. algae* Sa-BW1, Sa-BW2, Sa-BW7, Sa-BW8, and some other related *Shewanella* species. (a) Neighbor-joining tree. (b) Maximum-likelihood tree. GenBank accession numbers are given in parentheses.

Approximately 1400 bp nucleotide sequences of *16S rRNA* gene and 1100 bp nucleotide sequences of *gyrB* genes of *S. algae* ballast water isolates were used for phylogenetic analyses. GenBank nucleotide accession numbers for the *16S rRNA* and *gyrB* gene sequences are shown in the phylogenetic tree. A NJ phylogenetic tree based on *16S rRNA* (Figure-1a) indicated that the isolates fell within the clade comprising the members of genus *Shewanella*, forming a cluster with *S. algae* strain ATCC 51192 with sequences similarities of 99.4% (Sa-BW1), 99.9% (Sa-BW2), 99.8% (Sa-BW7), and 100% (Sa-BW8). The tree also revealed a very close phylogenetic relationship of the isolates with *Shewanella upenei* 20-23R with a same sequence similarities values as in *S. algae* ATCC 51192. The maximum-likelihood tree of *16S rRNA* gene (Figure-1b) showed almost the same topology except for the location of the nearest neighbor, *S. haliotis* DW01 which being clustered together with ballast water isolates, *S. algae* and *S. upenei*. The isolates shared sequences similarities of 98.9% (SA-BW1) and 99.2% (Sa-BW2, Sa-BW7, and Sa-BW8), to *S. haliotis* DW01 and 92.8-98.7% similarities to the other *Shewanella* species used in the phylogenetic analysis.

**Figure-2:**
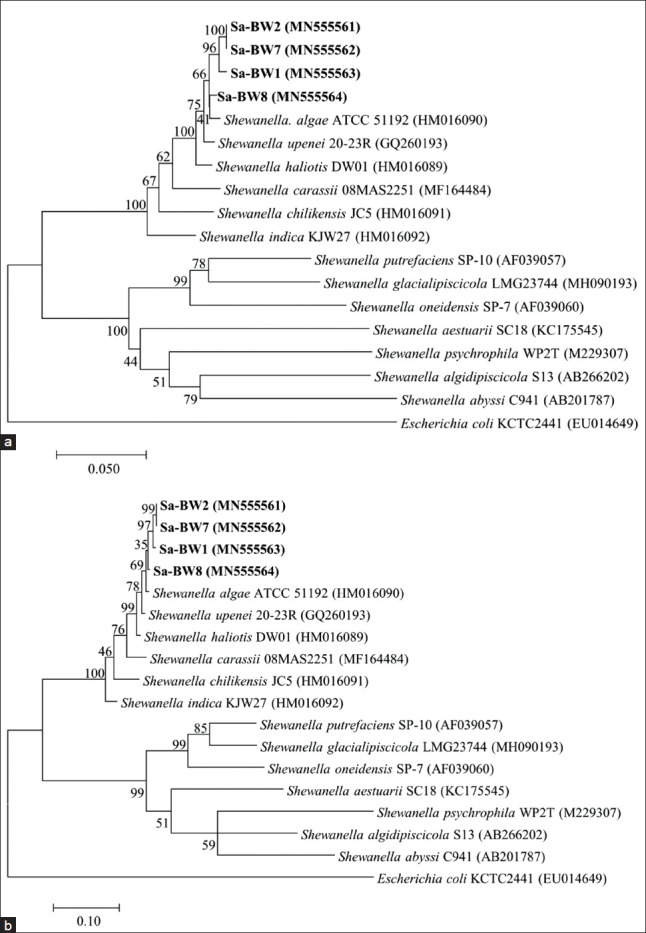
Phylogenetic tree based on *gyrB* gene sequences of *S. algae* Sa-BW1, Sa-BW2, Sa-BW7, Sa-BW8, and some other related *Shewanella* species. (a) Neighbor-joining tree. (b) Maximum-likelihood tree. GenBank accession numbers are given in parentheses.

However, the topology of NJ tree based on the *gyrB* gene (Figure-2a) showed that these isolates clustered monophyletically, apart from *S. upenei* and *S. haliotis*. The strains showed *gyrB* gene sequence similarities of 97.7-98.9% to *S. upenei*, 97.5-97.9% to *S. haliotis*, and 73.1-95.2% to other *Shewanella* species. Almost the same topology was observed in ML tree of *gyrB* gene (Figure-2b). Isolates Sa-BW2 and Sa-BW7 formed an independent cluster next to Sa-BW1 indicating that these two isolates have an almost identical sequence to each other with similarity values of 99.5%. Meanwhile, strain Sa-BW8 formed a separated branch which suggests the greater number of sequence difference from the other isolates. Based on phylogenetic analysis and sequence similarity data, strains Sa-BW1, Sa-BW2, Sa-BW7, and Sa-BW8 were clearly regarded as *S. algae*.

**Figure-3:**
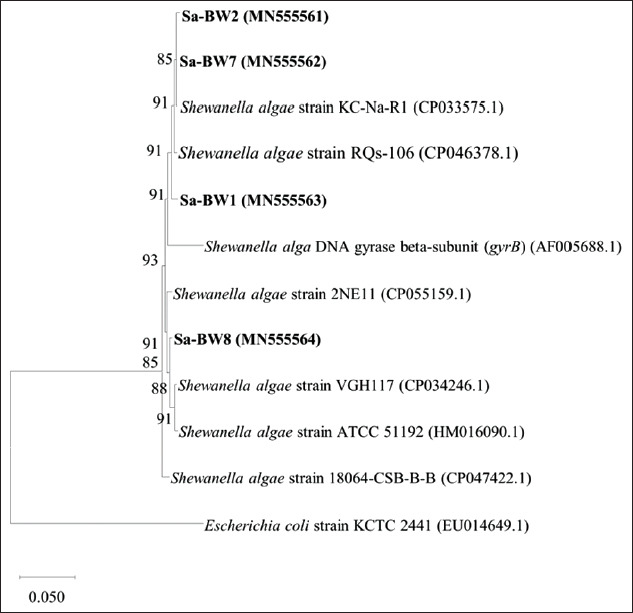
Maximum likelihood phylogenetic tree based on *gyrB* gene sequences of *Shewanella algae* Sa-BW1, Sa-BW2, Sa-BW7, Sa-BW8, and some other closely related *S. algae* strains. GenBank accession numbers are given in parentheses.

Further phylogenetic analysis of *S. algae* *gyrB* sequences from ballast water isolates against several nearest *S. algae* sequences from GenBank database allows for reasonable interpretation in determining the most likely origin of each isolates from the ballast tank. However, it is impossible to determine the exact origin of the bacteria found in ballast water. Based on the ML tree of *gyrB* gene generated in Figure-3, isolate Sa-BW1 which ballast water source was from Singapore port was closely related to *S. algae* strains RQs-106 from China. Isolate Sa-BW2 and Sa-BW7 isolates with source of ballast water from Yokkaichi port and Nhava Sheva port, respectively, were clustered together with *S. algae* strain KC-Na-R1 from South Korea. Meanwhile, isolate Sa-BW8 which sourced from Malacca Straits formed a separated branch closely related to strain 2NE11 from Peru. This result suggests that ballast water could transport bacterial species from various regions around the globe contributing to the global spread of the species.

### Biofilm formation capabilities

In this study, the cutoff OD_C_ was set as 0.143. The isolates that showed OD value lesser than 0.143 were considered as non-biofilm former while isolates with OD value more than 4 × OD_C_ were regarded as strong biofilm former. Comparing the OD means of each *S. algae* isolate to differentiation criterion; OD_c_ = 0.143, 2 × OD_c_ = 0.286, and 4 × OD_C_ = 0.332, all strains were observed to be strongly adherent with OD value; 1.31±0.9 (Sa-BW1), 1.23±1.0 (Sa-BW2), 1.16±0.5 (Sa-BW7), 0.91±0.4 (Sa-BW8), 0.93±0.01 (Sa-SW2), and 0.89±0.6 (Sa-SW3), respectively. Positive control, *S. aureus* showed OD value 0.92±0.05 indicated strong adherence capabilities.

### Antibiotic susceptibility

A similar pattern of antibiotic susceptibility was observed in all *S. algae* strains. As summarized in Table-3, multiple resistance was observed against oxacillin, ampicillin, carbenicillin, cephalothin, novobiocin, tobramycin, colistin, and rifampicin. Intermediate resistance to kanamycin, oxytetracycline, and streptomycin was also recorded. The ­isolates were only sensitive to ciprofloxacin, chloramphenicol and gentamicin. The MAR index recorded was >0.2 suggesting that the isolates were originated from a source that has antibiotic contamination [43].

**Table-3:** Antibiotic resistance profile of *S. algae* strains from ballast water.

Antibiotics	Disc content (μg)	Sensitivity
Ampicillin	20	R
Carbenicillin	100	R
Cephalothin	30	R
Ciprofloxacin	10	S
Chloramphenicol	30	S
Colistin	10	R
Gentamicin	10	S
Kanamycin	30	I
Novobiocin	30	R
Oxacillin	5	R
Oxytetracycline	30	I
Penicillin	10	R
Rifampicin	5	R
Streptomycin	25	I
Tobramycin	10	R
MAR index (a/b)		0.6

S = Susceptible, I = Intermediate, R = Resistance, a/b = calculation ratio. *S. algae*: *Shewanella algae*

## Discussion

In this study four *S*. *algae* was isolated from ballast water of ships docked in Port Klang, Malaysia. The isolates had key phenotypic characteristics of oxidase positive and sulfide production attributed to *Shewanella* spp. [18]. Additional phenotypic characteristics such as the ability to grow at 42°C, tolerance to high salt concentration (6-8%), and hemolysis on sheep blood agar distinguished the *S. algae* isolates from other species such as *S. putrefaciens*, *S. haliotis* and *S. xiamenensis* as described in the previous study [44]. These characteristics grouped the *S. algae* isolates into mesophilic and halophilic *Shewanella* strains as reported by several studies [45-47]. The biochemical characteristics of *S. algae* isolate from this study were similar to *S. algae* from the first Danish cases of *S. algae* bacteremia [48], shrimp (*P. vannamei*) [26], and type strain IAM 14159 [49]. Previous study by Altug *et al*. [4] also reported the presence of *S. algae* in several ballast water samples collected from ships berthed around Ambali Port, on the northern shores of Sea of Marmara.

The presence of *S. algae* in both ballast water and port water samples raises the possibility that some species may have been transported there through ballast water. Whether this species was introduced or is native and common in the local port water remains an open question. Since bacterial species may also be introduced to every marine environment in many ways and may be present in polluted environment, we cannot clearly conclude that the presence of *S. algae* in the local water is due to the release of ballast water. However, suppose the ballast water that contains the *S. algae* were released during cargo loading, the species will be transferred to local port water. The same goes to the species present in local water during unloading cargo process where ballasting procedure could transport the species into ballast tank to another port of call. These scenarios would validate the ballast water potential as a vector for global spread of microorganisms.

The phylogenetic analysis grouped the strains (Sa-BW1, Sa-BW2, Sa-BW7, and Sa-BW8) in the *S. algae* cluster, closest to *S. algae* ATCC 51192. Based on the comparative analysis of phylogenetic tree, the *gyrB* gene seems to be more reliable and useful than *16S rRNA* for describing phylogenetic relationship at the species level. The *S. algae* isolates showed independent branching from *S. haliotis* and *S. upenei* based on *gyrB* gene with similarities values of 97.0-97.9% and 97.7-98.5%, respectively, which were much lower than in *16S rRNA* (>99%). The lower occurrences of interspecies sequence similarities in *gyrB* gene compared to *16S rRNA* described in this study implied a better resolution to distinguish *S. algae* isolates from its closely related species, *S. haliotis* and *S. upenei*. It is known that the rate of molecular evolution of *gyrB* sequences is faster than *16S rRNA* which provides higher phylogenetic resolution. It was reported in several studies that *gyrB* gene has always been used as a discriminative detection for *Shewanella* species identification [50-52]. The similarities values of greater than 97% observed in both *gyrB* and *16S rRNA* sequences of *S. algae* isolates with *S. haliotis* and *S. upenei* revealed a very close phylogenetic association between the three species. This might also indicate a potential taxonomic problem in the presumptive identification of this related strain. However, in recent whole-genome sequencing *S. haliotis* and *S. upenei* were proposed as a later heterotrophic synonym of *S. algae* [53-55]. The phylogenetic analysis of *gyrB* gene sequence between S*. algae* isolates from ballast water and nearest *S. algae* strains sequences from GenBank database revealed the phylogenetically relatedness of the ballast water strain with *S. algae* strains from various region around the globe. The fact that the *S. algae* isolates from this study itself were isolated from ballast tanks of different ballast water sources (port of origin) would validate the role of ballast water in transporting the bacterial species around the world, contributing to the global spread. It can be difficult and almost impossible to track the exact origin of the bacterial species as ballast water exchange can be made at different ports [3]. In addition, the ballast tank can contain mixture of water from different ports because there is always a small portion of unpumpable water that remains before taking on cargo.

The capability of all *S. algae* isolates in this study to produce extracellular enzymes such as lipase, protease, DNase, and hemolysin could be inferred as part of adaptive mechanism for the species to survive in their respective marine environment. The expression of this phenotype could be aimed at reducing surrounding microbial competition or degrading organic matter to gain access to their nutrients [20,56]. However, the adaptation allowing survival in marine environment could also allow for colonization of living hosts if the bacteria get the opportunity to enter the host. These enzymes which are known to have pathogenic potential are capable of enhancing bacterial virulence as they enable the bacteria to breach and invade host tissue contributing to a wide range of infections [57-59]. According to Edberg *et al*. [60], no single extracellular enzyme has been proved to be the sole factor responsible for bacterial pathogenicity. Thus, it is considered necessary for microbes to contain more than one extracellular enzyme to be virulent. In general, hemolytic activity has been considered as an important virulence marker for *Shewanella* spp. to predict potentially virulent strain [61-63]. In a mouse pathogenicity study performed by Khashe and Janda [34], *S. algae* was observed to be the more virulent species compared to *S. putrefaciens*, and it was speculated that hemolytic activity could play an important virulence factor. Thus, the ability of *S. algae* isolates in this study to exhibit beta-hemolytic activity on sheep blood and human blood agar suggested their potential as putative pathogen.

The ability of *S. algae* isolates to form a strong biofilm formation, as shown in this study suggested a significant advantage for the survival adaptation of this species in ballast tank. The production of exopolysaccharides matrix in the formation of biofilms could provide protection from mechanical or chemical treatment and from predatory protists [64]. The biofilm environment may also promote phenotypic modification as well as genetic exchanges among the communities of microorganisms within biofilms [65]. Although it has not been tested, antibiotic resistance or virulence in *S. algae* may be enhanced through horizontal gene transfer should a novel genotype or toxigenic species arrive in ballast water. This protective film may act as refuge for bacteria during transport allowing them to persist within the tank environment and proliferate. In term of pathogenicity, formation of biofilms provides protection against immune system and antibiotic treatment thus preventing access of certain antimicrobial agents from reaching the bacterial cells within the biofilm which could complicate the clinical treatment of *S. algae* [66].

Resistance to antibiotics has been inferred to encourage host pathogenesis allowing persistent or chronic diseases [67]. Studies on antibiotic susceptibility profiles of *Shewanella* spp. indicated that most species are susceptible to aminoglycosides, carbapenems, erythromycin, quinolones, extended-spectrum cephalosporins, and macrolides, but resistance to penicillin [68-70]. However, there has been an increase in the occurrence of multidrug resistance in *S. algae* strains [71-73] including the isolates from the current study. The most commonly reported antibiotic resistance is against beta-lactams, such as amoxicillin, ampicillin, and penicillin; and against cephalosporins such as cephalothin, cefazolin, and cefotaxime [74] in accordance with the resistance observed in this study. Notably, *S. algae* is frequently reported to be resistant to colistin [73]. Resistance to tobramycin, novobiocin, and rifampicin was also observed in several studies [24,26,73]. The multiple resistance profile observed in *S. algae* strains of ballast water are of concern as horizontal gene transfer of antibiotic resistance genes might occur due to the closed system and water retention time within ballast tanks [75].

## Conclusion

In this study, four *S. algae* strains were isolated from ballast water samples taken from ships docked at Port Klang, Malaysia. The isolates were found to produce hemolytic activity on sheep blood agar, secreted several extracellular enzymes (lipase, protease, DNase, and gelatinase), performed a strongly adherent biofilm and demonstrated multiple resistances toward antibiotics. These characteristics may represent the putative pathogenic factor of the *S. algae* strains. The presence of putative pathogenic strains in ballast water suggested that ships carry a potential risk to local marine environment, should they release the pathogenic strains during ballasting operation. While *S. algae* is already present in Port Klang waters, further introduction of it could pose a risk to the local ecosystem. Hence, monitoring level of pathogenic species should be continued in incoming ballast water to protect the local environment from bacteriological risks and to guard the public on possible health risks in port environments.

## Authors’ Contributions

NNNI, NMN, and FS designed the study. NNNI and NMN did laboratory analysis and collected data. FKS, FS, and AA reviewed the manuscript. FKS and FS were the supervisors for the study. All authors read and approved the final manuscript.
